# Stability of large diffusion/perfusion mismatch in anterior circulation strokes for 4 or more hours

**DOI:** 10.1186/1471-2377-10-13

**Published:** 2010-02-10

**Authors:** R Gilberto González, Reza Hakimelahi, Pamela W Schaefer, Luca Roccatagliata, A Gregory Sorensen, Aneesh B Singhal

**Affiliations:** 1 Neuroradiology Division, Department of Radiology, Massachusetts General Hospital, Boston, Harvard Medical School, Boston, MA 02114, USA; 2 Department of Neurosciences, University of Genoa, Via De Toni, 5-16132 Genoa, Italy; 3 AA Martinos Center for Biomedical Imaging, Department of Radiology, Massachusetts General Hospital, Harvard Medical School, Boston, MA, USA; 4 Department of Neurology, Massachusetts General Hospital, Boston, Harvard Medical School, Boston, MA 02114, USA

## Abstract

**Background:**

The stability of hypoperfused brain tissue in stroke patients with major artery occlusions is unknown. The purpose of this study was to determine the persistence of a diffusion/perfusion mismatch in patients with ICA or proximal MCA occlusions.

**Methods:**

Fourteen patients with ICA and/or proximal MCA occlusion and a diffusion/perfusion mismatch at presentation were studied. All were enrolled in a pilot randomized study of normobaric oxygen therapy. None received thrombolytic therapy; 8 received normobaric oxygen and 6 room air. Diffusion/perfusion MRI was performed at baseline, 4 hours, 24 hours, and 1 week. Abnormal DWI, ADC, and MTT volumes were determined using standard image analysis methods.

**Results:**

The mean time from symptom onset to baseline MRI was 7.5 ± 1 hours. Across all 4 time points there was a significant difference in DWI lesion (ANOVA, P < 0.0001) and abnormal MTT volumes (ANOVA, P < 0.01) with the 24 hour and 1 week abnormal volumes different from the earlier studies. However, comparing baseline and 4 hour scans, there was no significant interval change in the mean abnormal DWI volume (29.4 ± 8.2 ml vs. 28.1 ± 7.4 ml) or abnormal MTT volumes (137 ± 17.7 ml vs. 130.9 ± 13.8). By 24 hours, only 2 patients did not maintain a mismatch of 20% or greater.

**Conclusions:**

Patients who present outside the time window for thrombolytic therapy, and who have a large diffusion/perfusion mismatch on MRI may have a stable mismatch for 4 or more hours.

## Background

The stability of the region of hypoperfused but still viable brain in patients with a cerebral artery occlusion is unknown. It is likely variable and dependent upon the quality of the collateral circulation, but this variability is largely unexplored. It is commonly assumed that the growth of the infarct core is rapid [1], and this is an important rationale for implementing thrombolytic therapy as quickly as is feasible [2]. The purpose of this study was to investigate the stability of diffusion and perfusion MRI abnormalities, in a series of patients with ICA or proximal MCA occlusions who were enrolled in a previously reported pilot study of normobaric oxygen therapy (NBO) and who were imaged at the time of study inclusion, then at 4 hours, 24 hours, and 1 week later [3].

## Methods

We analyzed data on 16 patients with ICA and/or proximal MCA occlusion enrolled in a pilot study [3] of normobaric oxygen therapy (NBO), approved by the Massachusetts General Hospital Human Research Committee (2001-P-001176). This randomized, placebo-controlled study with blinded MRI analysis had the following inclusion criteria:

1. Non-lacunar, anterior circulation ischemic stroke presenting <12 hours after witnessed symptom onset or <15 hours after last seen neurologically intact

2. Ineligible for intravenous/intra-arterial thrombolysis

3. National Institutes of Health Stroke Scale (NIHSS) score ≥ 4

4. Pre-admission modified Rankin scale (mRS) score ≤ 1

5. Mean transit time (MTT) lesion larger than DWI lesion (perfusion/diffusion "mismatch") with evidence for cortical hypoperfusion on MRI. To minimize time to treatment, "mismatch" was assessed during the initial MRI, using a visual estimate for >20% difference between DWI and MTT lesion size.

The exclusion criteria were:

1. Active chronic obstructive pulmonary disease

2. More than 3 L/min oxygen required to maintain peripheral arterial oxygen saturation (SaO2) >95% as per current stroke management guidelines [2]

3. Rapidly improving neurological deficits

4. Medically unstable

5. Pregnancy

6. Inability to obtain informed consent

7. Contraindication for MRI

Eligible patients gave consent and were randomized by opening sealed envelopes containing treatment allocation to the NBO group (humidified oxygen via simple facemask at flow rates of 45 L/min) or the control group (room air or nasal oxygen 1 to 3 L/min if necessary to maintain SaO2 >95%). NBO was stopped after 8 hours; however, nasal oxygen was continued if clinically warranted.

MRI scans were obtained at admission and repeated at 4 hours after starting gas therapy, then at 24 hours, and 1 week. National Institutes of Health Stroke Scale (NIHSS) was recorded at all time points. Modified Rankin Scale (mRS) was recorded 3 months after admission. An unblinded clinical investigator monitored patients during therapy.

### Imaging Techniques

Patients underwent MRI imaging on a 1.5-T (General Electric, Waukesha, Wisconsin) clinical MRI system. Sagittal T1, axial DWI with ADC maps, T2, fluid-attenuated inversion recovery (FLAIR), and gradient-echo sequences were performed at each time point. In addition, perfusion MRI and head MRA were obtained with the admission, 4 hour, and 24 hour MRI scans.

Diffusion-weighted images were acquired using a field of view of 220 mm, 23 slices, thickness of 6 mm, gap of 1 mm, TR of 7.5 seconds, TE of 99 ms, acquisition matrix 128 × 128, and with b = 0 s/mm2 and b = 1000 s/mm2 in 6 diffusion-gradient directions (number of averages = 3). Isotropic diffusion weighted images (DWI) and apparent diffusion coefficient (ADC) maps were automatically calculated. Fluid-attenuated inversion recovery and T2-weighted imaging were performed with a fast-spin echo sequence, resulting in TR/TE of 9000/85 ms, TI of 1750 ms, and 256 × 128 matrix; and performed with gradient-echo T2* imaging, resulting in TR/TE of 800/20 ms and 256 × 192 matrix. The fluid-attenuated inversion recovery and gradient-echo series had the following relevant parameters: 24-cm field of view, 7-mm-thick axial-oblique slices aligned with the anterior-posterior commissure, and 20 slices contiguous, interleaved, and co-localized.

Perfusion MRI images were obtained using the standard dynamic contrast method by injecting a bolus of gadolinium DTPA (0.1 mmol/kg dose via power injector) with gradient-echo echo planar imaging, 11 slices, field of view of 220 mm, TR of 1.5 s, TE of 54 ms; 46 measurements were made; matrix was 128 × 128. Mean transit time (MTT) maps were used as the perfusion metric, and it was computed using the singular value decomposition deconvolution method with an arterial input function chosen from the ipsilateral MCA [4]. The 3-dimensional time-of-flight MRA consisted of a single slab, ≈ 7-cm-thick, positioned over the circle of Willis, coplanar to the other slice prescriptions. The relevant imaging parameters were TR/TE = 39/6.9 ms, 25° flip-angle, field of view = 24 × 18 cm, with a matrix of 224 × 160 for an in-plane resolution of ≈ 1 mm, reconstructed to 92 axial images, 1.6-mm-thick with 0.8-mm overlap, for a total acquisition time of 3 minutes and 11 seconds. The MRA source images were postprocessed into maximum intensity projection images using standard software tools.

All images were subjected to a motion-correction algorithm and diffusion-tensor images were corrected for eddy-current distortions using FLIRT [5].

### Image Analysis

Manual MRI analysis was performed by two neuroradiologists (L.R., P.W.S.) blinded to clinical presentation, treatment group, clinical course, and medications. DWI, FLAIR, and MTT lesions were outlined by visual inspection on each axial slice using a commercially available image analysis program (ALICE; Perceptive Informatics, Waltham, Mass) to yield total lesion volumes for each patient. Infarction volumes were calculated from DWI images except for the 1-week time point when FLAIR images were used [6]. Identical volumes were outlined on the baseline and 4 hour scans and ADC values were determined for each patient. Relative ADC values (rADC) of the lesions were calculated as the ratio of ipsilateral ADC to contralateral ADC of a similar region.

The absolute volume of the DWI/PWI mismatch was calculated as their difference. The percent mismatch was calculated as (MTT-DWI)/DWI × 100%. A mismatch volume of zero was recorded for cases in which the MTT lesion was smaller than the DWI lesion.

### Statistical Analysis

All values are reported as percentage, mean ± standard error, or median. Data were evaluated for normal distribution using the Kolmogorov-Smirnov test. Repeated measures ANOVA was used to assess changes in lesion volumes across all 4 time points. Subsequently, significant differences in lesion volumes and mean rADC at two different time points were isolated using a paired t test. P values of less than 0.05 were considered statistically significant.

## Results

We present the results of a further analysis of a previously reported pilot study of normobaric oxygen therapy (NBO) [3]. A total of 16 patients were enrolled, and all had distal ICA and/or proximal MCA occlusions. Of the 16 subjects, 11 had MCA embolism from atrial fibrillation, 1 had cryptogenic MCA embolism, and 4 patients had artery to artery embolism to the MCA stem or distal ICA from the ipsilateral carotid artery. No patient received thrombolytic therapy; 8 were treated with NBO and 6 with room air (RA). Two patients were excluded from the evaluation because they developed acute post-ischemic hemorrhagic conversion unrelated to therapy on follow-up scans [7]. Demographic and clinical stroke measurements are shown in Table 1. The mean age of the 14 patients was 68 ± 4.6 years, mean time from symptom onset to initial MRI was 7.5 ± 1 hours (range, 1.7-14.4 hours), and the mean NIHSS was 13.4. Blood pressure was obtained at the time of each MRI scan. There was no significant change in mean arterial BP from baseline to 4 hours (p = 0.6, paired t-test). The initial diffusion and perfusion MRI scans were obtained shortly after presentation in the emergency department. Subsequent scans were acquired at a mean of 4 hours (range, 1.2 to 7.1 hours), 24.7 hours (range, 21.3 to 27.7 hours), and 6.4 days (range, 4 to 9.9 days) following the first scan. Perfusion data of two room air patients at the 4 hour time were not included because excessive motion artifact in one, and because a poor contrast bolus in the other resulted in images that could not be analyzed. DWI and MTT measurements were found to have a normal distribution using the Kolmogorov-Smirnov Test.

**Table 1:** Demographic and clinical data of individual patient

Individual Patient Data									
**Patient No**.	**Age**	**Sex**	**Time of First Scan after Stroke Onset**	**Intervention**	**Baseline NIHSS**	**4 Hour NIHSS**	**24 Hour NIHSS**	**1 week NIHSS**	**3 Month mRS**

1	77	F	1.7	NBO	18	15	4	4	6
2	53	M	3.4	RA	11	12	11	7	1
3	67	F	4.0	NBO	8	4	4	0	1
4	50	M	4.2	NBO	4	2	5	10	4
5	75	F	4.9	RA	11	15	18	17	4
6	49	M	6.5	RA	14	13	13	14	5
7	96	F	7.3	RA	12	12	16	14	4
8	51	M	7.3	NBO	12	11	8	2	1
9	81	F	7.5	RA	8	10	14	13	4
10	80	F	8.3	RA	21	26	26	23	5
11	88	F	10.3	NBO	12	4	4	5	1
12	37	M	12.9	NBO	16	12	11	7	1
13	72	F	13.1	NBO	19	15	16	12	4
14	79	F	14.4	NBO	22	-	-	22	5

**Figure 1:**
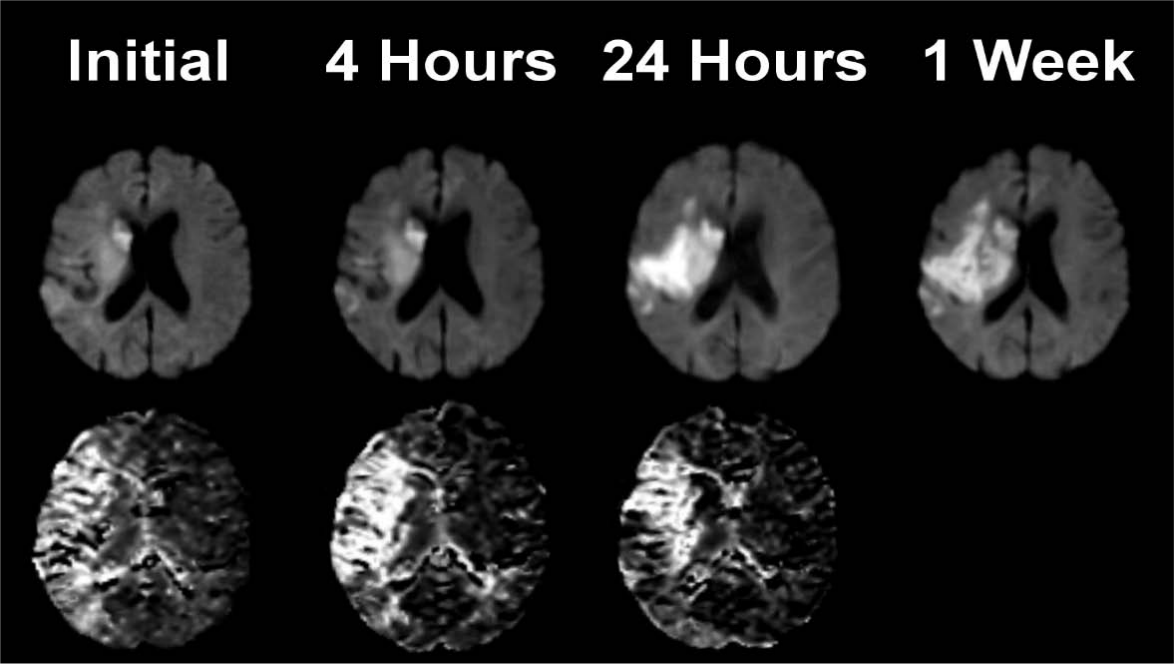
**Imaging data from a patient with a left hemiparesis**. Diffusion weighted images (top row) and mean transit time (bottom row) at the level of the lateral ventricles from a patient who presented 4.2 hours after onset of a left hemiparesis, and who was found to have a proximal right middle cerebral artery occlusion by CT angiography. Thrombolytic therapy was not given, but normobaric oxygen was provided by face mask for 4 hours. The image abnormalities were located within the right middle cerebral artery territory. The sizes of the diffusion and MTT abnormalities are very similar at the first 2 time points and there is a large mismatch. At 24 hours after baseline imaging, a large increase in the abnormal diffusion volume is apparent accompanied by a decrease in the abnormal MTT volume, but a greater than 20% mismatch persists. At 1 week, continued increase of the abnormal DWI is observed.

**Figure 2:**
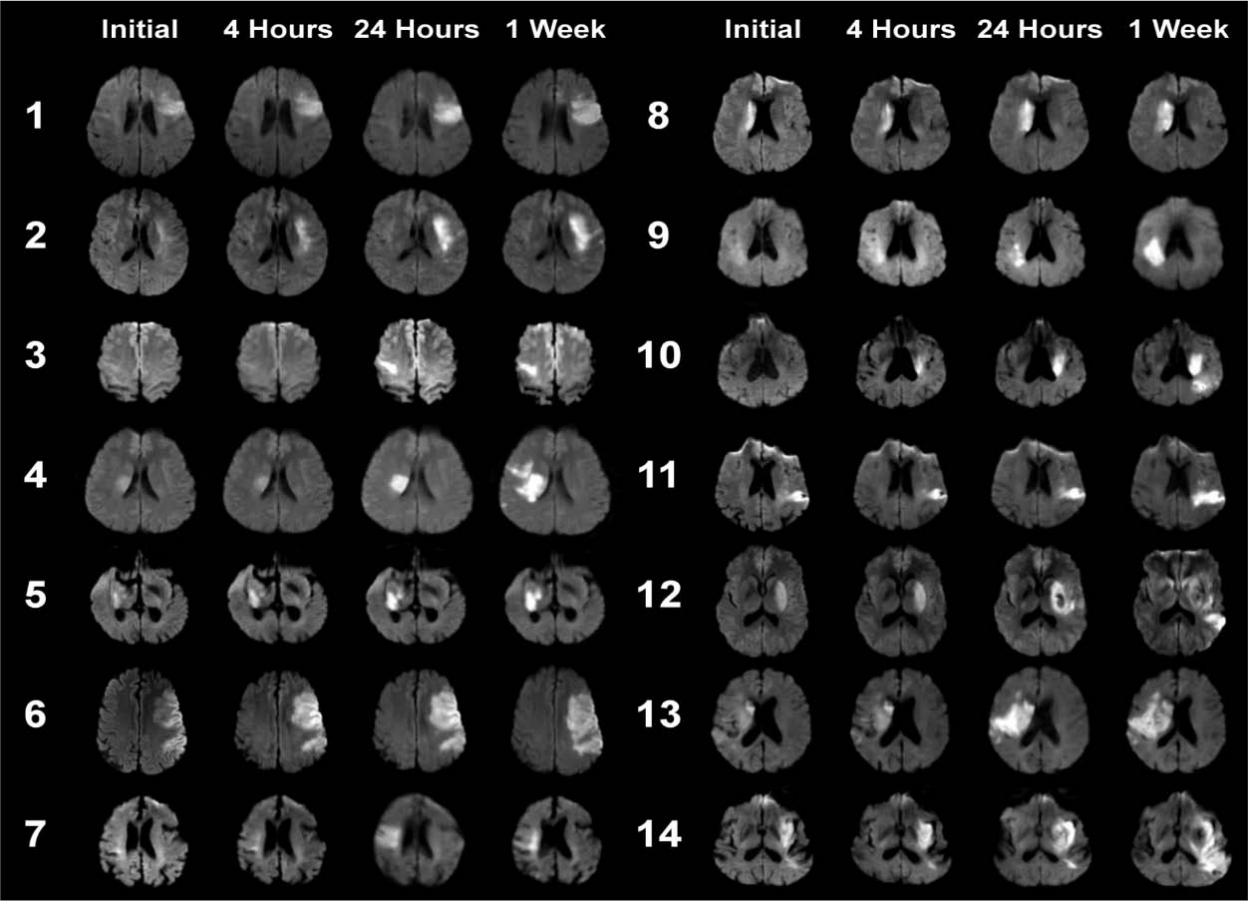
**Diffusion weighted images from all 14 patients**. Images were selected at the level of maximum lesion growth for each patient. The baseline, 4 hour, 24 hour and 1 week time points are displayed. The DWI hyperintense abnormalities did not change in most patients at 4 hours after baseline. However, there was an obvious increase in lesion volume by 24 hours after baseline in most patients, as well as at the 1 week time point.

An example of imaging data is shown in Figure 1. The DWI abnormality appears stable at 4 hours after the initial scan, but it increases substantially at 24 hours. The perfusion deficit is much larger than the DWI abnormality, and appears similar at baseline and 4 hours. It becomes smaller at 24 hours, but remains larger than the DWI lesion, indicating relative stability of the region of 'mismatch' over 24 hours. DWI images from each patient at the level of maximum growth are shown in Figure 2. DWI lesions did not change in most patients at 4 hours after baseline. However, most did show a clear increase in volume by 24 hours after baseline.

**Figure 3:**
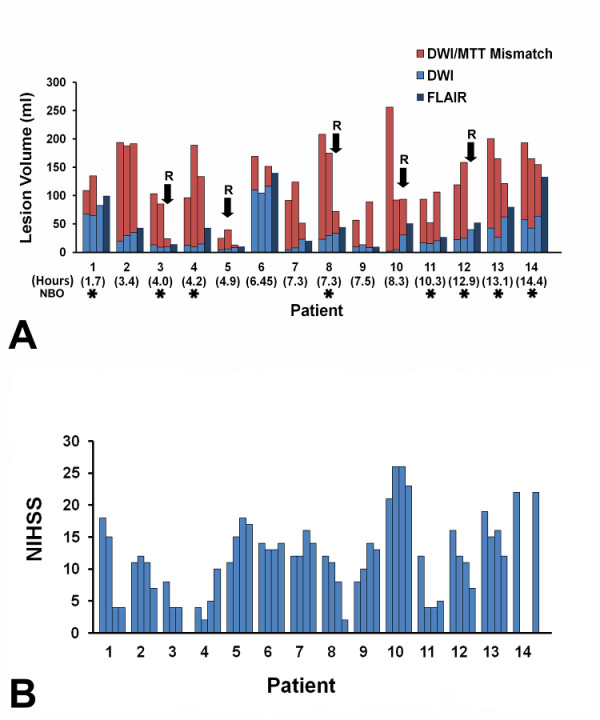
**DWI, FLAIR, and Mismatch volumes and NIHSS Scores of individual patients**. A. Abnormal DWI lesion volumes at baseline, 4 hours, and 24 hours are depicted in light blue;the 1-week FLAIR lesion volume is depicted in dark blue; and the red bars represent the 'Mismatch' lesion volumes (abnormal MTT lesion volume minus the abnormal DWI lesion volume). Five patients showed evidence for arterial recanalization (arrows, R) on serial MR angiography. One patient had partial recanalization at 4 hours, and four patients recanalized by 24 hours. The time of presentation in hours after stroke onset is shown in parenthesis below each patient number. Patients treated with NBO are marked with asterisks. Notably, a substantial volume of 'Mismatch' was maintained over the 24-hour period of imaging in most patients. B. The NIH stroke scale score for each patient at each imaging time point is displayed.

Abnormal DWI and MTT volumes from each patient are shown in Figure 3. At 4 hours after the initial scan, little change in DWI lesion volume is observed in most patients. At this time point only 1 patient (on room air) had more than 50% decrease in abnormal MTT volume, and 1 patient (receiving normobaric oxygen) had more than 30% decrease in abnormal MTT volume. By 24 hours, 5 patients (4 NBO and 1 RA) showed 50% or more decreases in abnormal MTT volume, and 3 other patients (1 NBO and 2 RA) had decreases of more than 30%.

**Figure 4:**
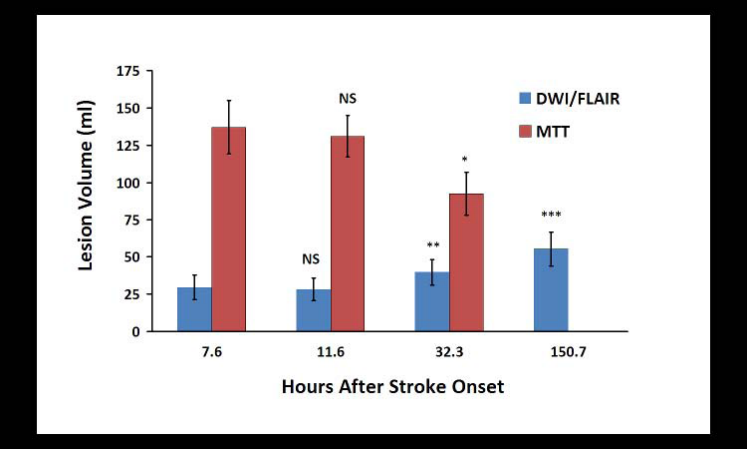
**Average abnormal DWI and MTT Volumes for all patients at each time point**. The mean abnormal DWI/FLAIR volumes for the baseline, 4 hour and 24 hour time points are shown in light blue. The red bars represent the abnormal MTT volume. The mean time in hours that the images were acquired after stroke onset is shown in parenthesis on the horizontal axis. Significant differences were found with respect to time point for abnormal DWI/FLAIR volumes (ANOVA, P < 0.0001) and MTT mismatch (ANOVA, P < 0.01). No differences in these measures were found between the baseline and 4 hour scans. Significant differences were isolated between the baseline and 24 hour DWI volumes (**, P < 0.001), between the baseline abnormal DWI and 1 week abnormal FLAIR volumes (***, P < .0001), and the baseline and 24 hour MTT volumes (*, P = 0.015). The mean MTT abnormality volumes remained substantially larger than the mean DWI abnormal volumes at the baseline, 4 and 24 hour time points.

The mean DWI volumes for each time point are illustrated in the Figure 4. Across all 4 time points there were significant differences (ANOVA, P < 0.0001). There was no significant interval change in the mean DWI abnormal volumes between baseline and the 4 hour scan (29.4 ± 8.2 and 28.1 ± 7.4 ml, P = 0.518). There was a slight decrease in DWI volume in patients that received NBO (32.5 ± 5.7 and 28.2 ± 5 ml, P = 0.155) and a slight increase in patients on room air at 4 hours (25.4 ± 11.2 and 28.1 ± 10.4 ml, P = 0.235). However, there were significant differences between baseline and 24 hours (29.4 ± 8.2 and 39.5 ± 8.5 ml, P = 0.001), and between 4 and 24 hours (28.1 ± 7.4 and 39.5 ± 8.5 ml, P = 0.002).

The mean rADC did not show any significant changes between baseline and 4 hour scans in RA group (0.72 ± 0.06 and 0.77 ± 0.07, P = 0.142). However, the mean rADC values increased significantly among NBO patients in the same period of time (0.64 ± 0.03 and 0.69 ± 0.04, P = 0.009).

All patients had substantial perfusion deficits estimated by prolonged MTT at baseline (Figure 3A). Across all 4 time points there was a significant difference (ANOVA, P < 0.01). However, there was no significant interval change in the mean abnormal MTT volumes between baseline and the 4 hour scan (137 ± 17.7 ml and 130.9 ± 13.8 ml, P = 0.586). There was a significant difference in mean abnormal MTT volume between baseline and 24 hours (137 ± 17.7 ml and 92.5 ± 14.4 ml, P = 0.015), and between 4 and 24 hours (130.9 ± 13.8 ml and 92.5 ± 14.4 ml, P = 0.012). The mean absolute DWI/PWI mismatch volume did not change between baseline and 4 hours. By 24 hours, recanalization was documented in 3 NBO patients (3, 8, and 12) and one RA patient (10) while only patient 5 (RA) had evidence of recanalization on MRA at 4 hour time point. There is no MRA data for patient 9. However, only 2 cases did not maintain a mismatch of 20% or greater. A mean of 28.5% of the observed mismatch volume measured at 24 hours was incorporated into the final infarct volume at the one week scan.

The neurological examination of most patients underwent minor changes between the baseline and 4 hour evaluations (Table 1 and Figure 3B). Only 2 patients had a change of more than 4 points on the NIHSS: 1 NBO patient in whom the NIHSS dropped from 12 at baseline to 4 at 4 hours, and 1 RA patient in whom the NIHSS increased from 21 to 26. At the 24 hour evaluation, 6 of 13 patients had substantial changes on the NIHSS, with 2 decreasing and 4 increasing 4 points or more. There was no statistically significant correlation between outcome, defined by mRS at 3 months, and any of the imaging and clinical measures obtained at baseline, 4 hours and 24 hours.

## Discussion

The maintenance of large diffusion/perfusion mismatches for 4 hours in 12 of 14 patients with proximal anterior circulation arterial occlusions, and the minor change in mean ADC values during this timeframe, was unexpected. While the potential neuroprotective effects of NBO [8,9] might account for some degree of stability, these results were observed across both arms of the study and more likely support a stable physiology. The striking stability in the sizes of diffusion and perfusion abnormalities differs from and challenges the commonly accepted concept of rapid growth of the infarct core in these patients [1], and points to potential opportunities for patient management. A much greater enlargement of the diffusion abnormality was observed at 24 hours, but large diffusion/perfusion mismatches persisted among a high proportion of these acute stroke patients.

Many studies have documented that without treatment the presence of a significant diffusion/perfusion mismatch will result in a final infarct size larger than the original DWI lesion. Indeed, several trials have used such a mismatch as a selection criterion for trials of novel stroke treatment. DWI images obtained using 1.5 T echo planar MRI scanners when the 'b' parameter is 1000 are robust with high reproducibility between scanners, and display high contrast between acute severely ischemic and normal parenchyma.

Aside from positron emission tomography measures, DWI lesion volumes are the best estimate of infarct core volumes available by imaging. While it is possible that in the present study infarct cores grew but MR imaging did not detect lesion growth, it is highly unlikely. The best data derived from animal studies have shown that diffusion abnormalities precede infarction, and it has never been reported that an early infarct is larger than the diffusion abnormality. In humans, the DWI volume in untreated individuals is the same or smaller than the final infarct as is the case for all of the patients in this study. The stability of the DWI abnormality was also supported by our finding that the ADC values of the lesions did not change at 4 hours after the initial scan.

While there is general agreement with respect to DWI, there is much more variability in the perfusion parameter employed. There are several parametric maps that may be generated by analyses of dynamic contrast MRI studies including cerebral blood volume (CBV), cerebral blood flow (CBF), mean transit time (MTT), time to peak (TTP), time to peak of the residue function (Tmax) [10], and others. The deconvolution method employed in the study here uses an arterial input function to derive MTT and CBV, and CBF is calculated (it is equal to CBV/MTT). MRI derived CBF values correlate highly with 15O-water positron emission tomography and have been used to identify penumbral flow [11]. The MTT analysis is the most sensitive for identifying tissues with altered hemodynamics including areas of true ischemia, benign oligemia and fully compensated tissue with normal CBF but prolonged transit times. The advantages are that MTT identifies the maximum extent of tissue possibly at risk and it provides very high image contrast compared normally perfused tissues (Figure 1), which allows reproducible segmentation by visual inspection.

The maintenance of a mismatch for such long periods suggests that these patients had a cerebral physiology that was favorable, specifically a substantial collateral circulation preventing the significant growth of the diffusion lesion [12,13]. The high proportion of patients that have such a favorable physiology is likely due to the inclusion criteria, specifically a requirement for a significant diffusion/perfusion mismatch by MRI. The observation of a large mismatch in patients first imaged an average of 7.6 hours after stroke onset despite a major artery occlusion suggests the presence of an excellent collateral circulation.

The actual proportion of patients with slow growing DWI lesion and stable diffusion/perfusion mismatches in the general acute stroke population is unknown, but may be higher than is generally assumed. In a study by Copen et al. [14] of 109 consecutive acute anterior circulation stroke patients who underwent diffusion/perfusion MRI while being evaluated in the emergency department, 68 had proximal artery occlusions. Of these 68 patients, 47 (68%) had DWI/PWI mismatches of 160% or greater. Interestingly, this degree of mismatch was found in 13 of the 19 patients who were scanned more than 9 hours after stroke onset. Other studies involving smaller cohorts reported similar results [15-17].

If it is confirmed that a substantial proportion of patients in the general population with acute proximal anterior circulation artery occlusions have diffusion/perfusion mismatches that are stable for hours, then several important clinical implications are apparent. Foremost, the time window for aggressive treatment of such patients may be very wide [18]. While our observations are preliminary and speculative with respect to the possible therapeutic implications, if diffusion/perfusion stability as observed in this study is confirmed, then it may be possible to pursue more time consuming, but more effective recanalization using endovascular approaches. It may be practical to transfer such patients from facilities that do not have recanalization capabilities to those sites that do [19].

However, there are caveats including, most importantly, the size of the DWI lesion. A patient with a DWI abnormality greater than 1/3 of the MCA territory is unlikely to benefit [18] from an endovascular intervention. Furthermore, the risk of hemorrhage as a result of this type of intervention is unknown in patients with a diffusion/perfusion abnormality that is of long duration. Thus, treatment conclusions from this small, preliminary study are premature and further studies are required.

Finally, the findings strongly suggest the need for additional care in patient selection for recanalization therapy that is not based solely on time, but also on arterial occlusion persistence and the presence of salvageable tissue. In particular, a short delay in those patients that would otherwise not be treated at all may be justified to perform additional diagnostic studies such as MRI. Of course, the time needs to be as short as possible.

## Conclusions

We conclude that a large proportion of patients who present outside the traditional time window for thrombolytic therapy, and who have a large diffusion/perfusion mismatch on MRI may have a cerebrovascular physiology permissive for a stable mismatch that may last 4 or more hours. The identification of such patients may allow sufficient time for well considered and definitive treatments.

## List of abbreviations

ADC: Apparent Diffusion Coefficient; ANOVA: Analysis of Variance; DTPA: Diethylenetriamine Penta-acetic Acid; DWI: Diffusion-weighted Imaging; FLAIR: Fluid-Attenuated Inversion-Recovery; FLIRT: FMRIB (Functional Magnetic Resonance Imaging of the Brain's) Linear Image Registration Tool; ICA: Internal Carotid Artery; MCA: Middle Cerebral Artery; MRA: Magnetic Resonance Angiography; MRI: Magnetic Resonance Imaging; mRS: Modified Rankin Scale; MTT: Mean Transit Time; NBO: Normobaric Oxygen; NIHSS: National Institutes of Health Stroke Scale; RA: Room Air; SaO2: Arterial Oxygen Saturation; TE: Echo Time; TR: Repetition Time.

## Competing interests

The authors declare that they have no competing interests.

## Authors' contributions

RGG conceived the study, analyzed and interpreted the data, and drafted the manuscript. RH performed ADC image analysis, analyzed and interpreted the data, and prepared the manuscript. PWS and LR performed DWI and MTT image analysis. PWS also made revisions to the manuscript. AGS obtained funding, assisted in data analysis and interpretation, and made critical revisions to the manuscript. ABS conducted the pilot clinical study, obtained funding, assisted in data analysis and interpretation, and made critical revisions to the manuscript.

All authors read and approved the final manuscript.

## Pre-publication history

The pre-publication history for this paper can be accessed here:

http://www.biomedcentral.com/1471-2377/10/13/prepub
